# Supplementary material for: Investigation of the General Molecular Mechanisms of Gallic Acid via Analyses of Its Transcriptome Profile
Source: Int J Mol Sci. 2024 Feb 15;25(4):2303. doi: 10.3390/ijms25042303 (PMC10888745; doi:10.3390/ijms25042303)
Supplement: Supplementary file 1 [file ijms-25-02303-s001.zip › ijms-2783215-supplementary.pdf]

Figure S1. Results of the protein-protein interaction (PPI) plot of potential targets. The PPI was created using the STRING-DB website (https://string-db.org/)

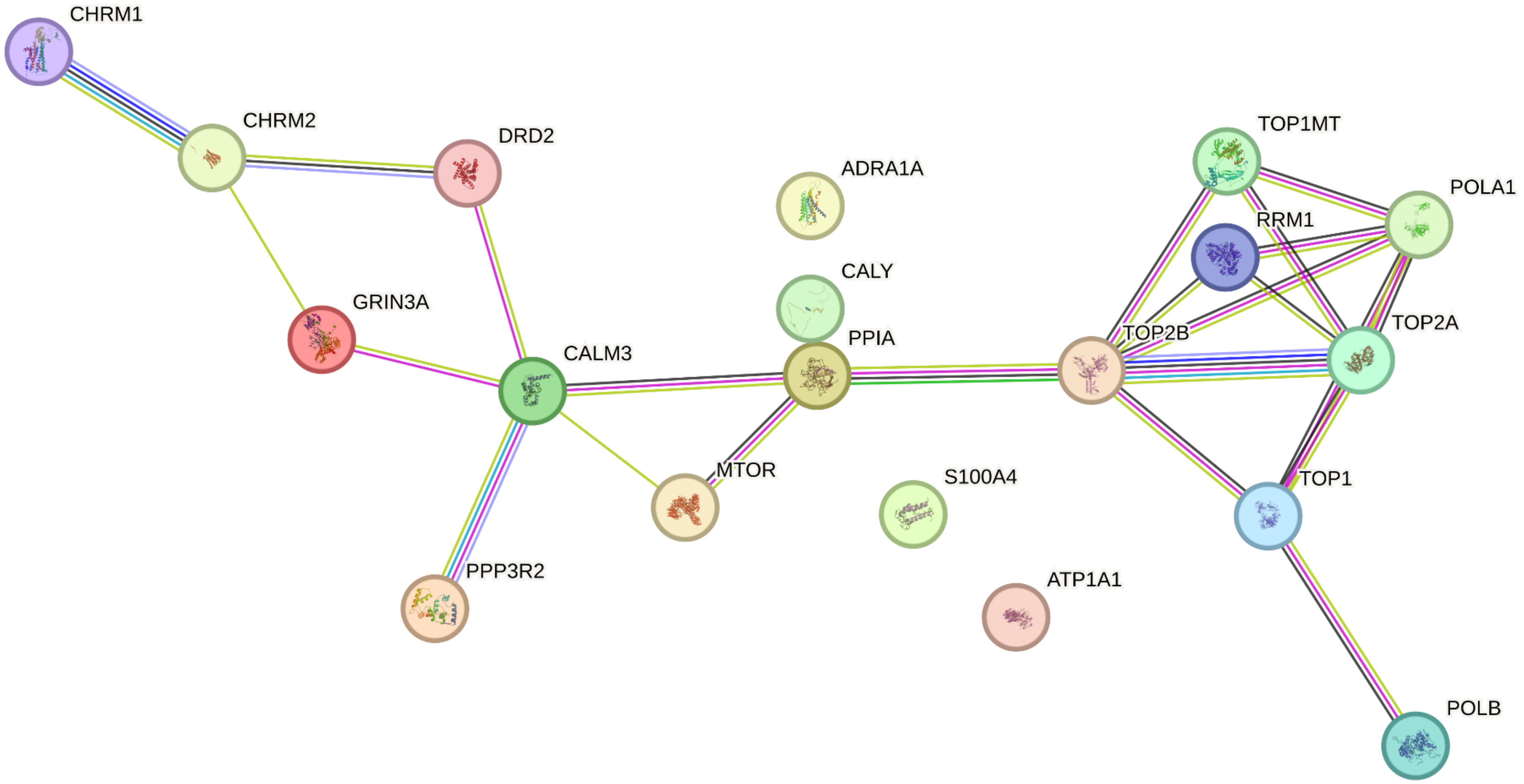

Table S1. Shared and unique genes from the Venn diagram

	Position (counts)	Genes
Upregulated genes	A549 (76)	*ANGPTL3 LRIG2 FMO2 GPR153 RERE YARS ZZZ3 POGZ CAPN8 KCNC4 TEX35 FSIP2 SCG2 LRRC58 IL17RD CP C3orf62 TLL1 RASSF6 SPOCK3 LPCAT1 PCDHB1 OXCT1 EXOC3 AIM1 HIST1H3G GNL1 MOXD1 ABHD16A AEBP1 LOC100130880 MACC1 LRRCC1 FAM110B SH3GL2 GCNT1 GK FTH1P18 TAF7L GLRA4 AMOT CT45A2 SRY OR13A1 CD6 CATSPER1 SERPINH1 LRRC32 PGA4 CLEC6A KRT71 C14orf28 SERPINA11 LOC646938 HERC2 GOLGA8R CHRNB4 GNPTG ITGAM GNAO1 HPR RPL3L ZNF646 KRT40 MYH14 KLK2 ZNF610 ZNF576 RBPJL GPCPD1 SNX5 CDH22 KCNG1 UMODL1-AS1 CACNG2 ZNF546*
	MCF7 (55)	*CD53 IBA57 RAB3B WDR78 ZBTB8B SNTG2 GALNT13 GCC2 HOXD10 KALRN CCR1 ACAP2 ZNF662 USP17L26 LDB2 SKP2 TCERG1 SLC6A7 UNC5A FOXQ1 ATXN1 PKHD1 SERPINE1 EN2 TMOD1 PRRG3 UBA1 PCDH11X TMLHE-AS1 ZNF22 LRRC18 SH3PXD2A NSUN6 LINC01219 ATG16L2 UBASH3B RFX4 LINC00934 SPERT PPP4R4 RNASE12 NUDT14 AAGAB FLOT2 FKBP10 TNFRSF11A SERPINB8 ZNF570 SIGLEC7 ZSCAN1 ADAMTS5 SLC19A1 PPARA RFPL3S LOC389834*
	PC3 (54)	*DIO1 LCE1D GJC2 GBP1 EFNA3 CNGA3 TP63 CLASP2 LSAMP PF4 HAND2 GPX3 JAKMIP2 TRIM40 FAM26F IYD CFAP69 ATP6V0A4 DEFB105B TRAPPC9 DEFA3 TLE4 KDM5C PJA1 IGSF1 SFTPA2 OR52B4 TEX40 DNAJC22 KIAA1033 AMIGO2 TAS2R30 DLK1 CYP1A2 ADAMTS17 ALDH1A2 VAT1L FOXL1 ATP6V0A1 SPHK1 ATPAF2 CDK12 SERPINB13 RNF125 GALP HAUS8 B9D2 MCEMP1 CEACAM21 ZNF773 LOC730668 CHEK2 DUSP16 PRAMEF9*
	A549 ∩ MCF7 (0)	
	A549 ∩ PC3 (0)	
	MCF7 ∩ PC3 (0)	
	A549 ∩ MCF7 ∩ PC3 (0)	
Downregulated genes	A549 (60)	*CSF3R OSCP1 VAV3 POGZ FAM129A DENND2C HK2 GLI2 APLF LRRC3B ARPP21 LARS2 TRAT1 C3orf80 CLCN2 RPL39L TRAPPC13 TTC1 SERINC5 TMEM170B SLC35B3 NHLRC1 LRFN2 TRAPPC3L HLA-DMB DGKB DEFB1 USP17L3 PDLIM2 FOXE1 IL3RA APEX2 TFE3 TSPY2 RPS4Y1 PLCE1 CUEDC2 ST5 TP53I11 C11orf68 ING4 LYRM5 C1RL RAD51 PARP6 NKD1 EMC8 RNF167 GRB7 TBC1D27 DTNA WDR83 USE1 SULT2B1 SUGP1 ZNF229 ZNF600 SOX12 TOX2 HNF4A*
	MCF7 (50)	*SPRR1B POGZ POTEE ARHGAP15 TRAT1 GRAMD1C CYP8B1 DNAH12 PF4V1 MND1 CRMP1 CCDC110 RNF180 PDE4D RIPPLY2 BVES BBS9 IGFBP1 MTRNR2L6 INTS9 RGS22 ZFPM2 MAGEE1 DOCK11 FRMD7 MRC1 CYP2C19 CNGA4 KBTBD3 OR6C2 MAP3K12 THAP2 CUL4A OR4K1 PPP2R3C GREM1 POTEB STOML1 TTC23 LYSMD4 ARHGAP23 OR1D5 RDM1 KRTAP4-1 LYRM9 ZNF844 UPK1A DGKD HMBS FMN1*

	PC3 (62)	*YBX1 ATXN7L2 MOV10 RGL1 OCLM PPAP2B RP5-1198O20.4 CYB5RL LOC101928327 NCKAP5 RBMS3 B4GALT4 AMIGO3 DCLK2 GRIA2 STOX2 BRD9 FAM13B COMMD10 AIF1 PRSS35 HUS1B TRERF1 KHDC1 WBSCR17 SP8 CAMK2B TECPR1 FAM122A OR5C1 POMT1 PRUNE2 CCBL1 LCNL1 KIF5B RP11-380G5.2 RHOD NRGN OR5M3 CNTF ZBED5 SOCS2 UBE3B TAS2R13 OR4K13 TMEM251 BBS4 CHTF8 RGS11 NFATC3 LOC100506388 KRTAP2-1 EFCAB13 CD226 SWSAP1 DUS3L ZNF577 PLCG1 PLCB1 APOBEC3F NDUFA6 FMN1*
	A549 ∩ MCF7 (2)	*TRAT1 POGZ*
	A549 ∩ PC3 (0)	
	MCF7 ∩ PC3 (1)	*FMN1*
	A549 ∩ MCF7 ∩ PC3 (0)	

Table S2. Gene list for each cell line from the GO enrichment analysis

	Cell line	Biological process (BP)	Counts	%	*p*-value	Genes
**Upregulated genes**	A549	GO:0006071~glycerol metabolic process	2	2.82	0.044682341	*GK, ANGPTL3*
		GO:0031638~zymogen activation	2	2.82	0.069326766	*HPR, KLK2*
		GO:0034765~regulation of ion transmembrane transport	3	4.23	0.070825417	*CATSPER1, KCNG1, KCNC4*
		GO:0010951~negative regulation of endopeptidase activity	3	4.23	0.082098247	*SERPINA11, SPOCK3, SERPINH1*
		GO:0007586~digestion	2	2.82	0.09334527	*CAPN8, PGA4*
	MCF7	GO:0006357~regulation of transcription from RNA polymerase II promoter	10	18.87	0.014104071	*FOXQ1, ZNF570, RFX4, EN2, ZNF22, LDB2, HOXD10, ZBTB8B, ZSCAN1, ZNF662*
		GO:0090026~positive regulation of monocyte chemotaxis	2	3.77	0.05108425	*CCR1, SERPINE1*
	PC3	GO:0042403~thyroid hormone metabolic process	2	3.92	0.020301791	*IYD, DIO1*
		GO:0061844~antimicrobial humoral immune response mediated by antimicrobial peptide	3	5.88	0.025546262	*DEFA3, GALP, PF4*
		GO:0097401~synaptic vesicle lumen acidification	2	3.92	0.035813066	*ATP6V0A4, ATP6V0A1*
		GO:0048485~sympathetic nervous system development	2	3.92	0.035813066	*HAND2, TP63*
		GO:0007035~vacuolar acidification	2	3.92	0.05108425	*ATP6V0A4, ATP6V0A1*
		GO:0009954~proximal/distal pattern formation	2	3.92	0.057556448	*ALDH1A2, TP63*
		GO:0032502~developmental process	2	3.92	0.057556448	*HAND2, TP63*
		GO:0008283~cell proliferation	3	5.88	0.066321132	*ALDH1A2, SPHK1, TP63*
		GO:0042771~intrinsic apoptotic signaling pathway in response to DNA damage by p53 class mediator	2	3.92	0.070371681	*CHEK2, TP63*
		GO:1900745~positive regulation of p38MAPK cascade	2	3.92	0.070371681	*HAND2, SPHK1*
		GO:0035115~embryonic forelimb morphogenesis	2	3.92	0.070371681	*ALDH1A2, TP63*
		GO:0090307~mitotic spindle assembly	2	3.92	0.089275813	*CHEK2, CLASP2*
		GO:0001568~blood vessel development	2	3.92	0.097556567	*ALDH1A2, SPHK1*
**Downregulated genes**	A549	GO:0051106~positive regulation of DNA ligation	2	3.57	0.010511038	*RAD51, APLF*
		GO:0006310~DNA recombination	3	5.36	0.020265091	*RAD51, POGZ, APEX2*

	GO:0045944~positive regulation of transcription from RNA polymerase II promoter	8	14.29	0.03851468	*TOX2, HNF4A, POGZ, TFE3, ZNF229, ZNF600, SOX12, GLI2*
	GO:0045859~regulation of protein kinase activity	2	3.57	0.051482703	*PLCE1, NHLRC1*
	GO:0031069~hair follicle morphogenesis	2	3.57	0.078681064	*FOXE1, GLI2*
MCF7	GO:0007165~signal transduction	8	16.00	0.016218424	*OR1D5, GREM1, IGFBP1, DGKD, TRAT1, PDE4D, ARHGAP15, ARHGAP23*
	GO:0007608~sensory perception of smell	3	6.00	0.023547553	*OR1D5, CNGA4, OR6C2*
	GO:0001782~B cell homeostasis	2	4.00	0.053735537	*DOCK11, PPP2R3C*
	GO:0002027~regulation of heart rate	2	4.00	0.071671591	*PDE4D, BVES*
PC3	GO:0021766~hippocampus development	3	5.77	0.012008849	*KIF5B, DCLK2, BBS4*
	GO:0001816~cytokine production	2	3.85	0.015825386	*NFATC3, CD226*
	GO:0035556~intracellular signal transduction	5	9.62	0.019297638	*SOCS2, DCLK2, RGS11, PLCG1, PLCB1*
	GO:0046488~phosphatidylinositol metabolic process	2	3.85	0.031405932	*PLCG1, PLCB1*
	GO:0007186~G-protein coupled receptor signaling pathway	6	11.54	0.062913743	*OR4K13, OR5M3, TAS2R13, RGS11, PLCB1, OR5C1*
	GO:0007165~signal transduction	7	13.46	0.065932287	*CAMK2B, GRIA2, CNTF, FAM13B, CD226, PLCB1, NRGN*
	GO:0032870~cellular response to hormone stimulus	2	3.85	0.066118975	*SOCS2, AIF1*
	GO:0051017~actin filament bundle assembly	2	3.85	0.078820393	*RHOD, AIF1*

Table S3. Gene list for each cell line from the KEGG pathway enrichment analysis

	Cell line	Pathway	Counts	%	*p*-value	Genes
**Upregulated genes**	A549					
	MCF7					
	PC3	hsa04145:Phagosome	3	5.88	0.072155	*SFTPA2, ATP6V0A4, ATP6V0A1*
		hsa04966:Collecting duct acid secretion	2	3.92	0.076161	*ATP6V0A4, ATP6V0A1*
		hsa05152:Tuberculosis	3	5.88	0.096456	*SPHK1, ATP6V0A4, ATP6V0A1*
**Downregulated genes**	A549	hsa04640:Hematopoietic cell lineage	3	5.36	0.028459	*CSF3R, HLA-DMB, IL3RA*
	MCF7	hsa04740:Olfactory transduction	4	8.00	0.059161	*OR1D5, OR4K1, CNGA4, OR6C2*
	PC3	hsa04728:Dopaminergic synapse	4	7.69	0.005011	*CAMK2B, GRIA2, KIF5B, PLCB1*
		hsa05022:Pathways of neurodegeneration - multiple diseases	6	11.54	0.00744	*CAMK2B, GRIA2, NDUFA6, KIF5B, PLCG1, PLCB1*
		hsa04360:Axon guidance	4	7.69	0.012122	*CAMK2B, NFATC3, PLCG1, RHOD*
		hsa04720:Long-term potentiation	3	5.77	0.013658	*CAMK2B, GRIA2, PLCB1*
		hsa05417:Lipid and atherosclerosis	4	7.69	0.018923	*CAMK2B, NFATC3, PLCG1, PLCB1*
		hsa04713:Circadian entrainment	3	5.77	0.027403	*CAMK2B, GRIA2, PLCB1*
		hsa04750:Inflammatory mediator regulation of TRP channels	3	5.77	0.027929	*CAMK2B, PLCG1, PLCB1*
		hsa05012:Parkinson disease	4	7.69	0.032903	*CAMK2B, NDUFA6, KIF5B, PLCG1*
		hsa04935:Growth hormone synthesis, secretion and action	3	5.77	0.040499	*SOCS2, PLCG1, PLCB1*
		hsa05016:Huntington disease	4	7.69	0.046823	*GRIA2, NDUFA6, KIF5B, PLCB1*
		hsa04723:Retrograde endocannabinoid signaling	3	5.77	0.058997	*GRIA2, NDUFA6, PLCB1*
		hsa04921:Oxytocin signaling pathway	3	5.77	0.063285	*CAMK2B, NFATC3, PLCB1*
		hsa04310:Wnt signaling pathway	3	5.77	0.075222	*CAMK2B, NFATC3, PLCB1*

Table S4. Merged gene list from the GO enrichment analysis

	Biological process (BP)	Counts	%	*p*-value	Genes
**Upregulated genes**	GO:0006357~regulation of transcription from RNA polymerase II promoter	12	57.14	2.15E-07	*FOXQ1, ZNF570, RFX4, HAND2, EN2, ZNF22, LDB2, HOXD10, ZBTB8B, TP63, ZSCAN1, ZNF662*
	GO:0045944~positive regulation of transcription from RNA polymerase II promoter	7	33.33	0.001075	*RFX4, HAND2, EN2, LDB2, HOXD10, TP63, PF4*
	GO:0061844~antimicrobial humoral immune response mediated by antimicrobial peptide	3	14.29	0.005601	*DEFA3, GALP, PF4*
	GO:0042403~thyroid hormone metabolic process	2	9.52	0.009274	*IYD, DIO1*
	GO:0048935~peripheral nervous system neuron development	2	9.52	0.011324	*HAND2, HOXD10*
	GO:0006071~glycerol metabolic process	2	9.52	0.014391	*GK, ANGPTL3*
	GO:0097401~synaptic vesicle lumen acidification	2	9.52	0.016431	*ATP6V0A4, ATP6V0A1*
	GO:0048485~sympathetic nervous system development	2	9.52	0.016431	*HAND2, TP63*
	GO:0007035~vacuolar acidification	2	9.52	0.023538	*ATP6V0A4, ATP6V0A1*
	GO:0009954~proximal/distal pattern formation	2	9.52	0.026569	*HOXD10, TP63*
	GO:0032502~developmental process	2	9.52	0.026569	*HAND2, TP63*
	GO:0031069~hair follicle morphogenesis	2	9.52	0.031601	*FOXQ1, TP63*
	GO:0042475~odontogenesis of dentin-containing tooth	2	9.52	0.060302	*HAND2, TP63*
	GO:0031640~killing of cells of other organism	2	9.52	0.070976	*DEFA3, PF4*
	GO:0050829~defense response to Gram-negative bacterium	2	9.52	0.092923	*DEFA3, GALP*
**Downregulated genes**	GO:0045944~positive regulation of transcription from RNA polymerase II promoter	10	33.33	4.57E-05	*GREM1, TOX2, HNF4A, POGZ, TFE3, NFATC3, ZNF229, ZNF600, SOX12, GLI2*
	GO:0007165~signal transduction	10	33.33	6.59E-05	*OR1D5, GREM1, IGFBP1, DGKD, TRAT1, PDE4D, CD226, ARHGAP15, PLCB1, ARHGAP23*
	GO:0035556~intracellular signal transduction	6	20.00	5.15E-04	*SOCS2, DGKD, DCLK2, RGS11, PLCG1, PLCB1*
	GO:0007608~sensory perception of smell	4	13.33	6.20E-04	*OR1D5, CNGA4, OR6C2, BBS4*

	GO:0021766~hippocampus development	3	10.00	0.005345	*KIF5B, DCLK2, BBS4*
	GO:0051106~positive regulation of DNA ligation	2	6.67	0.005987	*RAD51, APLF*
	GO:0006310~DNA recombination	3	10.00	0.006858	*RAD51, POGZ, APEX2*
	GO:0001816~cytokine production	2	6.67	0.010455	*NFATC3, CD226*
	GO:0046488~phosphatidylinositol metabolic process	2	6.67	0.020804	*PLCG1, PLCB1*
	GO:0010717~regulation of epithelial to mesenchymal transition	2	6.67	0.020804	*GREM1, APLF*
	GO:0045893~positive regulation of transcription, DNA-templated	5	16.67	0.022015	*HNF4A, TFE3, NFATC3, PLCB1, GLI2*
	GO:0050862~positive regulation of T cell receptor signaling pathway	2	6.67	0.023741	*TRAT1, CD226*
	GO:0032331~negative regulation of chondrocyte differentiation	2	6.67	0.035407	*GREM1, GLI2*
	GO:0009954~proximal/distal pattern formation	2	6.67	0.038302	*GREM1, GLI2*
	GO:0006357~regulation of transcription from RNA polymerase II promoter	7	23.33	0.039713	*TOX2, HNF4A, TFE3, NFATC3, ZNF229, ZNF600, GLI2*
	GO:0019216~regulation of lipid metabolic process	2	6.67	0.058336	*HNF4A, BBS4*
	GO:0008277~regulation of G-protein coupled receptor protein signaling pathway	2	6.67	0.068201	*RGS11, PLCB1*
	GO:0007173~epidermal growth factor receptor signaling pathway	2	6.67	0.071001	*DGKD, PLCG1*
	GO:0006281~DNA repair	3	10.00	0.071521	*RAD51, POGZ, APEX2*

Table S5. Merged gene list from the KEGG pathway enrichment analysis

	Pathway	Counts	%	*p*-value	Genes
**Upregulated genes**					
**Downregulated genes**	hsa05022:Pathways of neurodegeneration - multiple diseases	6	50.00	2.22E-04	*CAMK2B, GRIA2, NDUFA6, KIF5B, PLCG1, PLCB1*
	hsa04728:Dopaminergic synapse	4	33.33	6.11E-04	*CAMK2B, GRIA2, KIF5B, PLCB1*
	hsa04360:Axon guidance	4	33.33	0.001553451	*CAMK2B, NFATC3, PLCG1, RHOD*
	hsa05417:Lipid and atherosclerosis	4	33.33	0.002505658	*CAMK2B, NFATC3, PLCG1, PLCB1*
	hsa04720:Long-term potentiation	3	25.00	0.003445262	*CAMK2B, GRIA2, PLCB1*
	hsa05200:Pathways in cancer	5	41.67	0.003968449	*CAMK2B, CSF3R, IL3RA, PLCG1, PLCB1*
	hsa05012:Parkinson disease	4	33.33	0.004581751	*CAMK2B, NDUFA6, KIF5B, PLCG1*
	hsa04658:Th1 and Th2 cell differentiation	3	25.00	0.006404559	*HLA-DMB, NFATC3, PLCG1*
	hsa05016:Huntington disease	4	33.33	0.006781315	*GRIA2, NDUFA6, KIF5B, PLCB1*
	hsa04713:Circadian entrainment	3	25.00	0.007097647	*CAMK2B, GRIA2, PLCB1*
	hsa04750:Inflammatory mediator regulation of TRP channels	3	25.00	0.007240215	*CAMK2B, PLCG1, PLCB1*
	hsa04640:Hematopoietic cell lineage	3	25.00	0.007384094	*CSF3R, HLA-DMB, IL3RA*
	hsa04659:Th17 cell differentiation	3	25.00	0.008737437	*HLA-DMB, NFATC3, PLCG1*
	hsa04935:Growth hormone synthesis, secretion and action	3	25.00	0.010702674	*SOCS2, PLCG1, PLCB1*
	hsa04723:Retrograde endocannabinoid signaling	3	25.00	0.015975274	*GRIA2, NDUFA6, PLCB1*
	hsa04921:Oxytocin signaling pathway	3	25.00	0.017225647	*CAMK2B, NFATC3, PLCB1*
	hsa04630:JAK-STAT signaling pathway	3	25.00	0.01984924	*SOCS2, CSF3R, IL3RA*
	hsa04310:Wnt signaling pathway	3	25.00	0.020759506	*CAMK2B, NFATC3, PLCB1*
	hsa05415:Diabetic cardiomyopathy	3	25.00	0.028923368	*CAMK2B, NDUFA6, PLCB1*
	hsa04020:Calcium signaling pathway	3	25.00	0.039378894	*CAMK2B, PLCG1, PLCB1*

	hsa04730:Long-term depression	2	16.67	0.077606843	*GRIA2, PLCB1*
	hsa05014:Amyotrophic lateral sclerosis	3	25.00	0.082856423	*GRIA2, NDUFA6, KIF5B*
	hsa05031:Amphetamine addiction	2	16.67	0.088763328	*CAMK2B, GRIA2*
	hsa05010:Alzheimer disease	3	25.00	0.090888602	*NDUFA6, KIF5B, PLCB1*
	hsa05223:Non-small cell lung cancer	2	16.67	0.092454805	*KIF5B, PLCG1*
	hsa00562:Inositol phosphate metabolism	2	16.67	0.093682273	*PLCG1, PLCB1*
	hsa05214:Glioma	2	16.67	0.096132682	*CAMK2B, PLCG1*
	hsa04971:Gastric acid secretion	2	16.67	0.097355627	*CAMK2B, PLCB1*

Table S6. Results of the GO enrichment analysis of the potential targets

	Term	Count	%	*p*-value	Genes
**Biological process (BP)**	GO:0006265~DNA topological change	4	21.05	5.67E-08	*TOP2A, TOP2B, TOP1MT, TOP1*
	GO:2000300~regulation of synaptic vesicle exocytosis	5	26.32	1.62E-07	*CHRM2, GRIN3A, CALM1, DRD2, ADRA1A*
	GO:0006260~DNA replication	5	26.32	4.17E-06	*POLB, POLA1, RRM1, TOP1MT, TOP1*
	GO:0009410~response to xenobiotic stimulus	4	21.05	0.00137551	*ATP1A1, TOP1, DRD2, ADRA1A*
	GO:0045870~positive regulation of single stranded viral RNA replication via double stranded DNA intermediate	2	10.53	0.001861282	*TOP2A, TOP2B*
	GO:0048511~rhythmic process	3	15.79	0.002298593	*TOP2A, GRIN3A, MTOR*
	GO:0042493~response to drug	4	21.05	0.002375007	*ATP1A1, TOP1, DRD2, ADRA1A*
	GO:0001933~negative regulation of protein phosphorylation	3	15.79	0.002664496	*DRD2, PPIA, MTOR*
	GO:0007207~phospholipase C-activating G-protein coupled acetylcholine receptor signaling pathway	2	10.53	0.002790696	*CHRM2, CHRM1*
	GO:0000819~sister chromatid segregation	2	10.53	0.002790696	*TOP2A, TOP2B*
	GO:0007197~adenylate cyclase-inhibiting G-protein coupled acetylcholine receptor signaling pathway	2	10.53	0.007425518	*CHRM2, CHRM1*
	GO:0040016~embryonic cleavage	2	10.53	0.007425518	*TOP2A, TOP1*
	GO:0060134~prepulse inhibition	2	10.53	0.012039989	*GRIN3A, DRD2*
	GO:0007213~G-protein coupled acetylcholine receptor signaling pathway	2	10.53	0.013880098	*CHRM2, CHRM1*
	GO:0000731~DNA synthesis involved in DNA repair	2	10.53	0.013880098	*POLA1, RRM1*
	GO:0032516~positive regulation of phosphoprotein phosphatase activity	2	10.53	0.014798938	*CALM1, MTOR*
	GO:0071880~adenylate cyclase-activating adrenergic receptor signaling pathway	2	10.53	0.016634194	*DRD2, ADRA1A*
	GO:0000712~resolution of meiotic recombination intermediates	2	10.53	0.01755061	*TOP2A, TOP2B*
	GO:0002028~regulation of sodium ion transport	2	10.53	0.01755061	*ATP1A1, DRD2*
	GO:1901386~negative regulation of voltage-gated calcium channel activity	2	10.53	0.01846622	*PPP3R2, DRD2*
	GO:0055117~regulation of cardiac muscle contraction	2	10.53	0.020295023	*CALM1, ADRA1A*
	GO:0098664~G-protein coupled serotonin receptor signaling pathway	2	10.53	0.025762141	*CHRM2, CHRM1*
	GO:0060078~regulation of postsynaptic membrane potential	2	10.53	0.026670521	*CHRM1, GRIN3A*
	GO:0006974~cellular response to DNA damage stimulus	3	15.79	0.027268266	*TOP2A, POLB, MTOR*

	GO:0035307~positive regulation of protein dephosphorylation	2	10.53	0.028484883	*CALM1, PPIA*
	GO:0032148~activation of protein kinase B activity	2	10.53	0.028484883	*PPIA, MTOR*
	GO:0043278~response to morphine	2	10.53	0.028484883	*DRD2, MTOR*
	GO:0006281~DNA repair	3	15.79	0.030037327	*POLB, POLA1, RRM1*
	GO:0002027~regulation of heart rate	2	10.53	0.032104027	*CALM1, DRD2*
	GO:0006303~double-strand break repair via nonhomologous end joining	2	10.53	0.032104027	*POLB, POLA1*
	GO:0042220~response to cocaine	2	10.53	0.034810025	*DRD2, MTOR*
	GO:0007616~long-term memory	2	10.53	0.034810025	*DRD2, MTOR*
	GO:0001975~response to amphetamine	2	10.53	0.037508877	*CALM1, DRD2*
	GO:0008542~visual learning	2	10.53	0.047343815	*DRD2, MTOR*
	GO:0010507~negative regulation of autophagy	2	10.53	0.050009553	*ADRA1A, MTOR*
	GO:0007186~G-protein coupled receptor signaling pathway	4	21.05	0.055342211	*CHRM2, CHRM1, CALM1, ADRA1A*
	GO:0007187~G-protein coupled receptor signaling pathway, coupled to cyclic nucleotide second messenger	2	10.53	0.058844555	*CHRM2, CHRM1*
	GO:0042752~regulation of circadian rhythm	2	10.53	0.059723775	*TOP2A, MTOR*
	GO:0032922~circadian regulation of gene expression	2	10.53	0.064108248	*TOP1, DRD2*
	GO:0007059~chromosome segregation	2	10.53	0.068473407	*TOP2A, TOP1*
	GO:0007409~axonogenesis	2	10.53	0.080593783	*TOP2B, DRD2*
**Molecular function (MF)**	GO:0003918~DNA topoisomerase type II (ATP-hydrolyzing) activity	2	10.53	0.002844499	*TOP2A, TOP2B*
	GO:0003917~DNA topoisomerase type I activity	2	10.53	0.003790966	*TOP1MT, TOP1*
	GO:0046982~protein heterodimerization activity	4	21.05	0.005244098	*TOP2A, TOP2B, ATP1A1, ADRA1A*
	GO:0016907~G-protein coupled acetylcholine receptor activity	2	10.53	0.006625279	*CHRM2, CHRM1*
	GO:0003677~DNA binding	6	31.58	0.006819884	*TOP2A, POLB, TOP2B, POLA1, TOP1MT, TOP1*
	GO:0003682~chromatin binding	4	21.05	0.010945447	*TOP2A, TOP2B, POLA1, TOP1*
	GO:0005524~ATP binding	6	31.58	0.01223629	*TOP2A, TOP2B, RRM1, ATP1A1, TOP1, MTOR*
	GO:0008301~DNA binding, bending	2	10.53	0.017886551	*TOP2A, TOP1*
	GO:0003887~DNA-directed DNA polymerase activity	2	10.53	0.025326965	*POLB, POLA1*
	GO:0004993~G-protein coupled serotonin receptor activity	2	10.53	0.031793626	*CHRM2, CHRM1*
	GO:0005515~protein binding	17	89.47	0.032993439	*TOP2A, TOP2B, RRM1, CHRM1,*

					ATP1A1, ADRA1A, MTOR, POLB, POLA1, GRIN3A, PPP3R2, CALY, S100A4, TOP1, CALM1, DRD2, PPIA
	GO:0097718~disordered domain specific binding	2	10.53	0.035470646	*RRM1, CALM1*
**Cellular components (CC)**	GO:0099056~integral component of presynaptic membrane	4	21.05	1.67E-05	*CHRM2, CHRM1, DRD2, ADRA1A*
	GO:0098978~glutamatergic synapse	6	31.58	2.51E-05	*CHRM2, CHRM1, GRIN3A, CALY, DRD2, ADRA1A*
	GO:0045211~postsynaptic membrane	4	21.05	6.95E-04	*CHRM2, CHRM1, GRIN3A, DRD2*
	GO:0099055~integral component of postsynaptic membrane	3	15.79	7.58E-04	*CHRM2, DRD2, ADRA1A*
	GO:0043679~axon terminus	3	15.79	0.001489719	*CHRM2, CHRM1, DRD2*
	GO:0032991~macromolecular complex	5	26.32	0.002699747	*TOP2A, POLB, ATP1A1, CALM1, PPIA*
	GO:0005654~nucleoplasm	10	52.63	0.003861697	*TOP2A, POLB, TOP2B, POLA1, TOP1MT, S100A4, TOP1, CALM1, ADRA1A, MTOR*
	GO:0042734~presynaptic membrane	3	15.79	0.005718912	*CHRM2, CHRM1, DRD2*
	GO:0030425~dendrite	4	21.05	0.006988819	*CHRM2, CHRM1, DRD2, MTOR*
	GO:0098981~cholinergic synapse	2	10.53	0.010414313	*CHRM2, CHRM1*
	GO:0045202~synapse	4	21.05	0.010943185	*CHRM2, CHRM1, GRIN3A, DRD2*
	GO:0005635~nuclear envelope	3	15.79	0.014024534	*POLA1, RRM1, MTOR*
	GO:0005634~nucleus	11	57.89	0.018755187	*TOP2A, POLB, TOP2B, POLA1, TOP1MT, S100A4, TOP1, ALM1, ADRA1A, PPIA, MTOR*
	GO:0005887~integral component of plasma membrane	5	26.32	0.033754455	*CHRM2, CHRM1, CALY, DRD2, ADRA1A*
	GO:0005876~spindle microtubule	2	10.53	0.037687319	*POLB, CALM1*
	GO:0030315~T-tubule	2	10.53	0.039368092	*ATP1A1, ADRA1A*
	GO:0043025~neuronal cell body	3	15.79	0.042972076	*CHRM2, RRM1, GRIN3A*
	GO:0099061~integral component of postsynaptic density membrane	2	10.53	0.043557902	*CHRM1, GRIN3A*
	GO:0001673~male germ cell nucleus	2	10.53	0.045228989	*TOP2A, TOP1*
	GO:0000228~nuclear chromosome	2	10.53	0.046897318	*TOP2A, TOP1*

Table S7. Results of the KEGG pathway enrichment analysis of the potential targets

Term	Count	%	*p*-value	Genes
hsa04024:cAMP signaling pathway	6	31.58	3.26E-05	*CHRM2, CHRM1, GRIN3A, ATP1A1, CALM1, DRD2*
hsa04020:Calcium signaling pathway	5	26.32	7.56E-04	*CHRM2, CHRM1, PPP3R2, CALM1, ADRA1A*
hsa04022:cGMP-PKG signaling pathway	4	21.05	0.003147047	*PPP3R2, ATP1A1, CALM1, ADRA1A*
hsa04080:Neuroactive ligand-receptor interaction	5	26.32	0.003628544	*CHRM2, CHRM1, GRIN3A, DRD2, ADRA1A*
hsa05031:Amphetamine addiction	3	15.79	0.006819104	*GRIN3A, PPP3R2, CALM1*
hsa04970:Salivary secretion	3	15.79	0.011876143	*ATP1A1, CALM1, ADRA1A*
hsa04728:Dopaminergic synapse	3	15.79	0.02351894	*CALY, CALM1, DRD2*
hsa04261:Adrenergic signaling in cardiomyocytes	3	15.79	0.029829002	*ATP1A1, CALM1, ADRA1A*
hsa05010:Alzheimer disease	4	21.05	0.030421681	*CHRM1, PPP3R2, CALM1, MTOR*
hsa04218:Cellular senescence	3	15.79	0.032068752	*PPP3R2, CALM1, MTOR*
hsa05034:Alcoholism	3	15.79	0.044654064	*GRIN3A, CALM1, DRD2*
hsa05167:Kaposi sarcoma-associated herpesvirus infection	3	15.79	0.047717809	*PPP3R2, CALM1, MTOR*
hsa05022:Pathways of neurodegeneration - multiple diseases	4	21.05	0.052420161	*CHRM1, PPP3R2, CALM1, MTOR*
hsa05170:Human immunodeficiency virus 1 infection	3	15.79	0.055944249	*PPP3R2, CALM1, MTOR*
hsa05163:Human cytomegalovirus infection	3	15.79	0.062181602	*PPP3R2, CALM1, MTOR*
hsa05030:Cocaine addiction	2	10.53	0.086000314	*GRIN3A, DRD2*
